# Accuracy of Ultrasound Elastography and Fibrosis-4 Index (FIB-4) in Ruling Out Cirrhosis in Obese Non-Alcoholic Fatty Liver Disease (NAFLD) Patients

**DOI:** 10.7759/cureus.29445

**Published:** 2022-09-22

**Authors:** Sofi Damjanovska, Daniel B Karb, Alok Tripathi, Jessica Asirwatham, Sarah Delozier, Jaime A Perez, Yngve Falck-Ytter, Stanley Cohen

**Affiliations:** 1 Internal Medicine, University Hospitals Cleveland Medical Center, Cleveland, USA; 2 Gastroenterology and Hepatology, University Hospitals Cleveland Medical Center, Cleveland, USA; 3 Gastroenterology, University Hospitals Lakehealt Medical Center, Mentor, USA; 4 Clinical Research Center, University Hospitals Cleveland Medical Center, Cleveland, USA; 5 Gastroenterology and Hepatology, Veterans Affairs Northeast Ohio Healthcare System, Case Western Reserve University, Cleveland, USA; 6 Hepatology, University Hospitals Cleveland Medical Center, Cleveland, USA

**Keywords:** non-alcoholic fatty liver disease, fibrosis-4 score, liver fibrosis, cirrhosis, transient elastography (fibroscan), steatosis nash

## Abstract

Introduction: Non-alcoholic fatty liver disease (NAFLD) is the most common cause of advanced liver disease in the USA. Liver biopsy, the gold standard diagnostic test for evaluating liver fibrosis, is associated with significant risk and expense. The accuracy of ultrasound elastography and Fibrosis-4 index (FIB-4) in the obese NAFLD population is unknown. We aimed to compare the accuracy of ultrasound elastography and FIB-4 to liver biopsy in ruling out cirrhosis in NAFLD patients at a tertiary, transplant referral center in the US.

Methods: We retrospectively evaluated 93 patients with a mean age of 53 years (SD: 13 years) who underwent liver ultrasound elastography and liver biopsy, and additionally calculated their FIB-4 at the time of biopsy. We compared the liver stiffness measurement (LSM) obtained from ultrasound elastography and FIB-4 with the pathology results for ruling out cirrhosis.

Results: 85% of the patients were white, 53% were female, average BMI was 34.7 (SD: 6.7), 52% had diabetes, and 53% had hypertension. For biopsy-proven cirrhosis (prevalence 15%), a cut-off value of 12.5 kilopascals (kPa) for F4 had a sensitivity of 92% and a specificity of 54%. Values below this threshold excluded cirrhosis with 98% certainty. Compared to FIB-4, ultrasound elastography showed higher accuracy in ruling out cirrhosis (92% vs. 80% sensitivity, 98% vs. 95% negative predictive value (NPV), respectively).

Conclusion: To our knowledge, this is the first study in a tertiary transplant referral center in the USA to show that ultrasound elastography was superior to FIB-4 and can be used as a reliable screening test to rule out cirrhosis in obese NAFLD patients at a 12.5 kPa cut-off. Therefore, helping to avoid the risk and expense associated with liver biopsy.

## Introduction

Non-alcoholic fatty liver disease (NAFLD) is defined as evidence of hepatic steatosis with the lack of additional causes of secondary hepatic fat accumulation (alcohol abuse, hereditary disorders etc.) [1,2]. NAFLD is the most common cause of advanced liver disease in the USA. The overall prevalence is estimated to be 24%, with Hispanic Americans having the highest prevalence [3]. The inflammatory subtype of NAFLD is known as non-alcoholic steatohepatitis (NASH), which can progress to cirrhosis in 20% of patients, end-stage liver disease, and/or necessitating liver transplantation [4]. Additionally, NASH increases the risk of developing hepatocellular carcinoma (HCC), with an annual incidence of 0.7 to 2.6% among those with NASH cirrhosis [5].

With the growth of global obesity and diabetes rates, especially in the USA, the burden of disease associated with NAFLD, and NASH will continue to grow. NASH prevalence is projected to increase by 56% between the years of 2016 and 2030 in China, France, Germany, Italy, Japan, Spain, the United Kingdom, and the USA [6]. In 2017, lifetime healthcare costs of all US NASH patients was estimated to be $222.6 billion, a figure that is expected to increase [7].

The American Association for the Study of Liver Disease (AASLD) recommends liver biopsy to be performed in patients with NAFLD who are at increased risk of having steatohepatitis and/or advanced fibrosis [1]. The NASH Clinical Research Network (CRN) published the NAFLD activity score (NAS) to provide a standard measure of histologic changes assessed in NAFLD clinical trials [8]. The majority of published NASH literature describes fibrosis based on criteria from NASH CRN. NAS incorporates fibrosis stage and identification of NASH pattern recognition [9]. However, liver biopsy is an invasive procedure with risks and limitations. These include cost, sampling error, biopsy-related morbidity (0.3%; pain, bleeding, infection, bile leak, and damage to other organs), and mortality (<0.01%) [10].

An alternative to biopsy is liver ultrasound elastography, a noninvasive bedside tool that uses liver stiffness measurement (LSM) to detect the degree of fibrosis. Ultrasound elastography is approved by the US Food and Drug Administration (FDA) for use in both adults and children with liver disease [10]. Studies show that liver ultrasound elastography is an accurate and reliable tool to detect moderate to severe fatty liver, although diagnostic performance of liver ultrasound elastography in patients with different degrees of adiposity to histology are lacking. The American Gastroenterological Association (AGA), as well as the AASLD, make no recommendation regarding a LSM cut-off value in assessment of cirrhosis in NAFLD patients [1,11,12]. 

Another simple, inexpensive, noninvasive test is Fibrosis-4 index (FIB-4). FIB-4 combines standard laboratory values (platelets, alanine transaminase (ALT), and aspartate transaminase (AST)) and age to estimate a patient’s degree of fibrosis without biopsy. FIB-4 was initially developed as a noninvasive test to stage liver disease in patients with human immunodeficiency virus (HIV) and hepatitis C virus (HCV) co-infection [13]. A FIB-4 value of 1.3 has been validated in patients with NAFLD [14-17].

The accuracy of liver ultrasound elastography and FIB-4 in obese NAFLD patients at tertiary transplant referral centers in the USA with high prevalence rate of cirrhosis has not been sufficiently examined. By comparing ultrasound elastography and FIB-4 to biopsy, we aimed to characterize the performance of these diagnostic tests in this important population.

## Materials and methods

We identified 180 patients suspected to have NAFLD who underwent liver ultrasound elastography followed by liver biopsy at a tertiary transplant referral center in Cleveland, Ohio, between 2010 and 2019 under the institutional review board (IRB)-approved protocol. We excluded 87 patients due to greater than moderate alcohol consumption (more than one drink a day for women and more than two drinks a day for men) and those with additional pathologies that can lead to hepatic steatosis (hepatitis C, hepatitis B, primary biliary cirrhosis (PBC), primary sclerosing cholangitis (PSC), autoimmune hepatitis, alpha-1 antitrypsin deficiency, hemochromatosis, and drug-induced liver injury (DILI)), which left us a study population of 93 patients. FIB-4 was calculated not earlier than one month prior to liver biopsy. A FIB-4 cut-off below 1.45 and below 1.3 was used to rule out advanced fibrosis. LSM was performed using transient elastography (FibroScan, Echosens, France) and an extra large (XL) probe as part of standard clinical care for evaluation of liver fibrosis within a mean of eight months of liver biopsy. 10 consecutive and successful measurements were performed on each patient and an interquartile range (IQR) less than 30% were considered reliable. LSM was expressed in kilopascals (kPa). Controlled attenuation parameter (CAP) was expressed in decibel per meter (dB/m) and was obtained during transient elastography. The accuracy of ultrasound elastography and FIB-4 was determined by comparing LSM with pathology results obtained from liver biopsy. NASH CRN scoring was used to provide the grade of histologic changes, with fibrosis (F) grade 0 referring to no fibrosis present, F1 referring to presence of perisinusoidal or periportal fibrosis, F2 referring to presence of perisinusoidal and portal/periportal fibrosis, F3 referring to presence of bridging fibrosis, and F4 referring to presence of cirrhosis. The area under the receiver operating characteristic curve (AUROC) of the LSM value, as well as FIB-4, was calculated and used to determine the optimal cut-off value for F4. The accuracy of FIB-4 below 1.3 cut-off value in ruling out cirrhosis was assessed against the pathology results obtained from liver biopsy as well. NAS score (the sum of scores from steatosis, lobular inflammation, and ballooning; ranges from 0 to 8) was collected from the histologic assessment of the liver biopsy. The grading of the severity of steatosis on liver biopsy was collected (S0: fewer than 5% of hepatocytes are affected; S1: 5% to 33% of hepatocytes are affected; S2: 34% to 66% of hepatocytes are affected; and S3: more than 66% of hepatocytes affected). Electronic medical records (EMRs) were reviewed for demographic information, BMI, International Classification of Disease (ICD)-9/10 diagnosis of diabetes mellitus, anti-diabetic therapy, statin use, ICD-9/10 diagnosis of hypertension, dialysis requirement, medications, and laboratory values.

A series of Pearson and point biserial correlations were run as appropriate to assess the correlation between variables. All tests were two-tailed and p < 0.05 considered significant. Analysis was performed using R version 3.5.1 and GraphPad Prism.

## Results

We identified 93 NAFLD patients with a mean of 53 years of age (SD: 13 years) who underwent liver biopsy for a mean of one year (SD: 2 years) after ultrasound elastography. 85% of the patients were white, 53% were female, mean BMI was 34.7 (SD: 6.7), 52% had diabetes, and 53% had hypertension (Table 1). 13 patients had F0 stage on biopsy, 18 had F1 stage on biopsy, 23 had F2 stage on biopsy, 23 had F3 stage on biopsy, and 13 had F4 stage on biopsy. Four patients had biopsy reports that did not quantify the level of fibrosis. Prevalence of cirrhosis in this population was 15%. The severity of steatosis was reported for 87% of the patients and was mild (S0-S1: fewer than 5% to 33% of hepatocytes affected) in 33% of patients, moderate (S2: 34% to 66% of hepatocytes are affected) in 32%, and severe (S3: more than 66% of hepatocytes affected) in 19%.

**Table 1:** Study Cohort Characteristics SD: Standard deviation; kPa: Kilopascal; CAP: Controlled Attenuation Parameter; DM: Diabetes mellitus; HTN: Hypertension

	NAFLD
Number	93
Age (years) Mean (SD)	52 (13)
Race Black (n) (%)	7 (7.5%)
Race White (n) (%)	79 (85%)
Race Hispanic (n) (%)	3 (3%)
Race Other (n) (%)	4 (4.5%)
Sex Male (n) (%)	44 (47%)
Sex Female (n) (%)	49 (53%)
Fibroscan (kPa) Mean (SD)	15 (10)
Fibroscan (CAP) Mean (SD)	322 (53)
BMI Mean (SD)	34.7 (6.7)
DM Type2 (n) (%)	48 (52%)
Metformin (n) (%)	30 (32%)
GLP-1 agonist (n) (%)	5 (5.4%)
Statin (n) (%)	38 (41%)
HTN (n) (%)	49 (53%)
FIB-4 Mean (SD)	1.67 (1.16)

**Figure 1:**
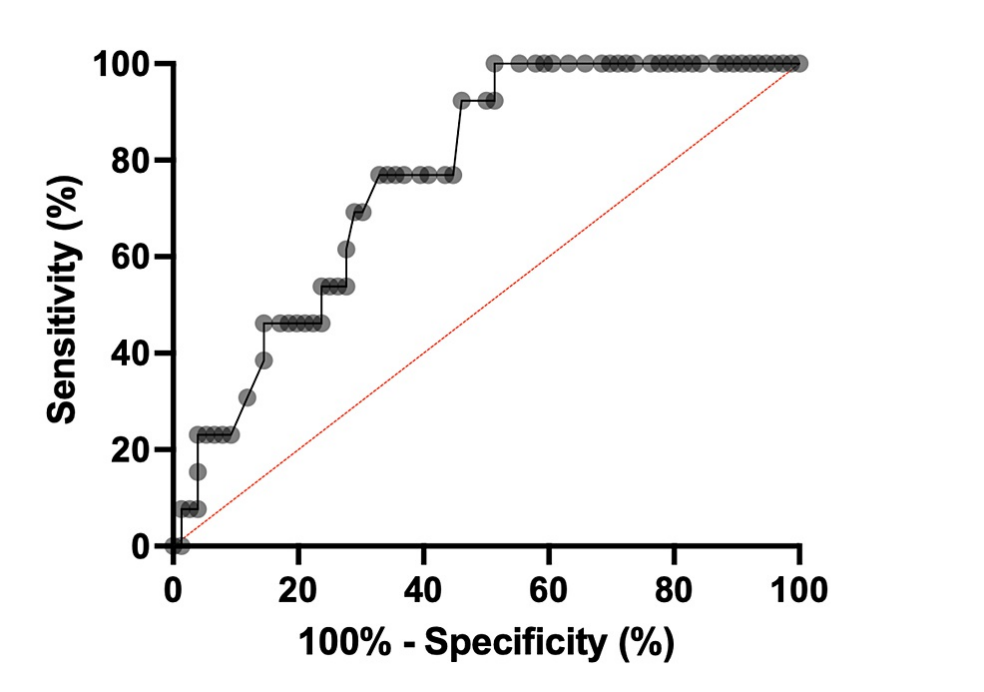
Receiver operating curve showing the performance of liver stiffness measurement (LSM) using transient elastography for ruling out cirrhosis (F4).

The AUROC for liver ultrasound elastography in the detection of F4 was 0.77 (95% CI, 0.66-0.88) (Figure 1). A cut-off value of 12.5 kPa for F4 revealed a sensitivity of 92% and a specificity of 54% (Table 2). Liver stiffness values below this threshold excluded cirrhosis (negative predictive value (NPV)) with 98% certainty. Only one patient with cirrhosis, who had an LSM of 11.9 kPa, would have been misdiagnosed with noncirrhotic fibrosis according to this cut-off value. 

**Table 2:** Comparison of the accuracy of transient elastography to FIB-4 at a 12.5 kPa cut-off for ruling out cirrhosis. *N=89, four patients had indeterminate F stage on biopsy. **N=72, four patients had indeterminate F stage on biopsy and 17 patient were lacking some of the laboratory values needed to calculate FIB-4. TP: True positive; FP: False positive; FN: False negative; TN: True negative; PPV: Positive predictive value; NPV: Negative predictive value

Non-invasive test	Cut-off	Prevalence of cirrhosis (%)	BMI	TP	FP	FN	TN	sensitivity (%)	specificity (%)	PPV	NPV
Transient elastography*	12.5	15	34.7 +/- 6.7	12	35	1	41	92	54	26	98
FIB-4**	<1.45	15	34.7 +/- 6.7	7	28	2	34	78	55	20	94
FIB-4**	<1.3	15	34.7 +/- 6.7	7	31	2	31	78	50	18	94

**Figure 2:**
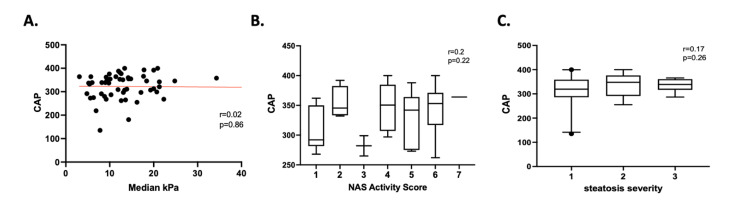
(A) Simple linear regression between CAP and median kPa, both measured with transient elastography; (B) CAP distribution in patients with certain NAS collected from the histologic analysis of the liver biopsy; (C) CAP distribution in patients with certain steatosis severity from the histologic analysis of the liver biopsy. CAP: Controlled attenuation parameter; kPa: Kilopascal.

CAP measured during liver ultrasound elastography showed no correlation with the median kPa (r = 0.02, p = 0.86), the NAS activity score (r = 0.2, p = 0.22), or the severity of steatosis (r = 0.17, p = 0.26) acquired from the histological assessment of the liver biopsy (Figure 2).

The AUROC for FIB-4 in the detection of F4 was 0.67 (95% CI, 0.5-0.85). A cut-off value of 1.45 for FIB-4 had a sensitivity of 78% and a specificity of 55%. FIB-4 values below this threshold excluded cirrhosis (NPV) with 94% certainty (Table 2). Two patients with cirrhosis would have been misdiagnosed with noncirrhotic fibrosis according to this cut-off value. A cut-off value of 1.3 for FIB-4 had a sensitivity of 78% and a specificity of 50%. FIB-4 values below this threshold excluded cirrhosis with 94% certainty (Table 2).

## Discussion

In obese patients, NAFLD is the most common chronic liver disease that can lead to cirrhosis and HCC. Liver biopsy, an invasive procedure with significant risk and expense, is the reference standard for determining degree of liver fibrosis and diagnosing cirrhosis. The limitations of biopsy have led to the development of non-invasive estimates of liver fibrosis, including liver ultrasound elastography and FIB-4. While the performance of ultrasound elastography has been evaluated in the hepatitis C population, its accuracy for other disorders, particularly in obese NAFLD patients, has not been extensively studied. This is at least partly due to the well-documented technical limitations of ultrasound elastography in patients that are obese. There are multiple confounding factors that can increase the liver stiffness value on ultrasound elastography (inflammation, fatty infiltration, cholestasis, congestive hepatopathy, or a postprandial increase in blood flow) [18-22]. Furthermore, when performing liver ultrasound elastography, the technician must choose between a standard M probe and an XL probe for overweight and obese patients, who have a significant amount of intercostal fat. When an M probe is used in obese patients, 19% will produce an uninterpretable result [18, 23-26]. Because of these, several studies in the past have excluded obese patients (BMI >/= 30 kg/m^2^). 

In a non-obese population with a low prevalence of cirrhosis, there is literature to suggest that LSM cut-off of 11.3-11.8 kPa excluded cirrhosis with 99% certainty [27]. To our knowledge, this study is the first from a tertiary referral center to evaluate liver ultrasound elastography in a cohort of NAFLD patients with a high percentage of obesity and cirrhosis prevalence. In this unique population, which may be more typical of a liver transplant center than a community practice, a LSM cut-off of 12.5 kPa had a 92% sensitivity for F4 fibrosis and excluded cirrhosis with 98% certainty. Only one patient with cirrhosis in this cohort, who had an LSM of 11.9 kPa, would have been misdiagnosed with noncirrhotic fibrosis. Although ultrasound elastography was also associated with a high rate of false positives, these data suggest that it can reliably be used to 'rule out' cirrhosis in obese, NAFLD patients.

Another strength of these data, as they pertain to the diagnostic characteristics of ultrasound elastography, is that they correlate liver biopsy to a wide range of LSM measurements, thus avoiding a potential selection bias whereby patients with low LSM are never referred for biopsy. Here, nearly 50% of subjects had LSM below 12.5 kPa. Additionally, we attempted to use this cohort to establish the utility of CAP, a measure of steatosis obtained from ultrasound elastography. CAP was not reliably correlated with LSM (derived from ultrasound elastography), NAS (derived from histology), or steatosis grade (derived from histology). The clinical utility of CAP remains unclear. When we compared liver ultrasound elastography to FIB-4, liver ultrasound elastography showed better accuracy for the exclusion of advanced fibrosis irrespective of the value we used. The FIB-4 value of below 1.45, which was obtained with the help of an AUROC had inferior sensitivity and NPV. As this value has been often described in the literature as a value used in HCV/HIV co-infection, we also looked at the value of below 1.3, as this value has been used and reported in the literature in patients with NAFLD [13, 15-17]. In our patient population the sensitivity and NPV stayed the same when compared to the below 1.45 value.

These findings are subject to a few limitations. First, this sample was collected from a larger cohort of patients with suspected NAFLD who underwent both liver ultrasound elastography and biopsy, it may not be standard clinical practice to obtain both ultrasound elastography and biopsy on patients with high clinical suspicion of NAFLD, and this study does not take into account any other factors which might have led to the decision to obtain liver biopsy. Second, ultrasound elastography and histologic analysis are both subject to inaccuracy due to sampling error, interobserver and intraobserver variability [28,29]. Third, because the data were obtained from EMRs, they are subject to the limitations of retrospective chart review. Lastly, another limitation is the small sample set.

Overall, this study adds to the body of evidence suggesting that ultrasound elastography has a valuable clinical role in NAFLD. Liver ultrasound elastography is safe, quick, and the cheapest radiographical test to assess for advanced fibrosis. With the ongoing development of several novel pharmacologic therapies for NAFLD, it is more important than ever to identify the patients that may be candidates for therapy, ideally with a non-invasive method. This study suggests that liver ultrasound elastography in obese NAFLD patients at a LSM cut-off value of 12.5 kPa can reliably rule out cirrhosis and can thus decrease exposure in these patients to unnecessary further workup, including liver biopsy. By the same token, the large number of false positives (n = 35) suggests that ultrasound elastography is best used only to exclude cirrhosis, and that a diagnosis of cirrhosis is less reliably established with a positive result. Ultrasound elastography was superior to both FIB-4 values we looked at in both sensitivity and NPV (92% vs. 78%; 98% vs. 94%, respectively). The clinical utility of CAP scores obtained during transient elastography examinations remains uncertain and requires further research. 

In this study of an obese population of NAFLD patients with a high prevalence of cirrhosis, we showed that ultrasound elastography with a 12.5 kPa LSM cut-off was highly accurate in ruling out cirrhosis. This was confirmed by liver biopsy results. Additionally, ultrasound elastography showed a better accuracy than two FIB-4 values in ruling out cirrhosis. These results suggest that this non-invasive study can be reliably used to rule out cirrhosis in obese NAFLD patients.

## Conclusions

NAFLD is the most common cause of advanced liver disease in the USA. To our knowledge, this is the first study in a tertiary transplant referral center in the USA to show that ultrasound elastography was superior to FIB-4 and can be used as a reliable screening test to rule out cirrhosis in obese NAFLD patients at a 12.5 kPa cut-off. Therefore, by using ultrasound elastography, providers can decrease exposure of obese NAFLD patients to unnecessary further workup, including liver biopsy that comes with its risks and expense.
